# Clinical Characteristics of Patients Seeking Treatment for Common Mental Disorders Presenting With Workplace Bullying Experiences

**DOI:** 10.3389/fpsyg.2020.583324

**Published:** 2020-11-05

**Authors:** Sarah Helene Aarestad, Ståle Valvatne Einarsen, Odin Hjemdal, Ragne G. H. Gjengedal, Kåre Osnes, Kenneth Sandin, Marit Hannisdal, Marianne Tranberg Bjørndal, Anette Harris

**Affiliations:** ^1^Department of Psychosocial Science, University of Bergen, Bergen, Norway; ^2^Department of Psychology, Norwegian University of Science and Technology, Trondheim, Norway; ^3^Diakonhjemmet Hospital, Oslo, Norway

**Keywords:** workplace bullying, common mental disorders, sick leave, health complaints, work

## Abstract

**Background:**

Targets of workplace bullying tend to develop severe mental health complaints, having increased risk of sick leave and expulsion from the workplace. Hence, these individuals are likely to be overrepresented among patients seeking treatment for common mental disorders (CMD). This study investigated the prevalence of exposure to workplace bullying in a patient group seeking treatment for CMD. Further we explored if exposed and non-exposed patients differed on clinical and work-related characteristics.

**Methods:**

The sample comprised of 675 patients from an outpatient clinic in Norway and consisted of 70% women and had a mean age of 39 (*SD* = 10.5) years. The study had a cross-sectional design and differences between the patient groups were analysed using chi-square, Mann–Whitney *U*-tests and independent sample *t*-tests.

**Results:**

The prevalence of exposure to bullying was 25.8%. The patients exposed to bullying reported significantly more major depressive disorders (MDDs) measured with the MINI psychiatric interview, higher levels of depressive symptoms, anxiety symptoms, subjective health complaints, alcohol use, and lower resilience as measured with questionnaires. Twice as many were on full-time sick leave, reported lower work ability, lower return to work self-efficacy, and lower job satisfaction. A majority preferred another job than the one they have today over returning to their current employment.

**Conclusion:**

Victims of workplace bullying are a vulnerable group at risk of expulsion from working life, being overrepresented among patients seeking mental health treatment for CMD. One in four patients represented with such experience have higher levels of psychological symptoms and are more often diagnosed with depression as compared to other patients. Thus, this is a problem that should be addressed in clinical settings. If not addressed there is an increased risk of sick leave and permanent exclusion from working life.

## Introduction

Common mental disorders (CMD) represents one of the leading causes of long-term sick leave (Organization for Economic Co-operation and Development (OECD), 2015), accounting for roughly 20% of those on sick leave and one third of disability pensions in the Norwegian working population (Organization for Economic Co-operation and Development (OECD), 2015; Brage and Nossen, 2017). While such sick leave is caused by a range of factors on multiple levels, several studies have established a strong link between exposure to workplace bullying, mental health and absenteeism from work (Lahelma et al., 2012; Einarsen and Nielsen, 2014; Verkuil et al., 2015; Magee et al., 2017). Among possible psychosocial work-related factors, exposure to workplace bullying has been established as a major risk factor for sick leave (Slany et al., 2014; Nielsen et al., 2016), as well as of expulsion from the workplace, and potentially from working life itself (Berthelsen et al., 2011; Glambek et al., 2015). Based on previous research, individuals exposed to workplace bullying represent a group with seemingly high levels of mental health complaints. Moreover, if considering the increased risk of sick leave and expulsion from the workplace, one could postulate that these individuals would be highly represented among patients seeking treatment for CMD. Hence, it is important to study the prevalence of workplace bullying in this population of patients and to explore how these individuals may or may not differ from other patients with CMD. To the best of our knowledge, only one study Tatar and Yüksel (2019) has yet investigated prevalence rates of workplace bullying in a clinical sample consisting of patients with CMD. However, Tatar and Yüksel (2019) included patients who had experienced bullying or other forms of psychological trauma at the workplace, thus we still lack knowledge of the prevalence of workplace bullying in the population of patients on sick leave or at risk due to CMD.

Exposure to bullying is a prevalent problem in contemporary working life that can be found across all professions and industries with prevalence rates in the area of 5 to 20%, depending on country, operational definitions and estimation methods (e.g., Nielsen et al., 2010; Zapf et al., 2020). Workplace bullying can be described as a long-term process where the target is subjected to systematic and unwanted negative behaviours at work, be it from superiors or peers (Einarsen et al., 2011). The exposure to these negative and unwanted behaviours can vary in both intensity and duration as bullying typically escalates over time (Einarsen, 2005). The said behaviours may be direct or indirect, verbal or non-verbal and typically of an either work-related or person-related nature, often involving some degree of social exclusion. In addition, there is often a power imbalance in the bully-victim relationship leading targets to experience difficulties defending themselves (Einarsen and Skogstad, 1996; Harvey et al., 2009).

Exposure to workplace bullying has been established as a major source of distress and subsequently been identified as an important contributory factor to severe health problems in the working population (e.g., Kostev et al., 2014; Verkuil et al., 2015). A growing body of evidence has established that being exposed to such bullying tend to have a range of detrimental effects on victims, hence also being a major work-related predictor of mental, psychosomatic and to some extent physical health problems (Vartia, 2001; Nielsen et al., 2012; Lever et al., 2019). Symptoms include negative health conditions such as, cardiovascular disease (Jacob and Kostev, 2017; Xu et al., 2018), musculoskeletal pain (Høgh et al., 2011; Kääriä et al., 2012; Buhaug et al., 2020), gastrointestinal symptoms (Lever et al., 2019), sleep difficulties (Hansen et al., 2014; Verkuil et al., 2015; Lever et al., 2019; Rodríguez-Muñoz et al., 2020), symptoms of post-traumatic stress (Mikkelsen and Einarsen, 2002; Tatar and Yüksel, 2019), and general stress (Vartia, 2001), in addition to being associated with an increase in CMD (Verkuil et al., 2015; Finstad et al., 2019; Lo Presti et al., 2019; Rodríguez-Muñoz et al., 2020).

Several longitudinal studies have shown that CMD, and other negative health outcomes, persist over time even long after ones exposure to workplace bullying (Lahelma et al., 2012; Nielsen and Einarsen, 2012). For instance, a study by Bonde et al. (2016) found that depressive disorders and sick leave resulting from exposure to bullying persisted over several years, regardless of whether the bullying had ceased or not. Considering these facts, it is very likely that individuals who have experienced bullying will need, seek, and receive treatment for their health problems. It is therefore of value to investigate what characterises these individuals and also to examine if their symptoms are of greater or lesser severity or differ from those patients without bullying experience. Such information should be of great value when assessing these patients’ treatments needs, when designing treatment procedures and in helping them in order to be able to return to work and avoiding expulsion from work and working life.

The purpose of the present study was therefore to investigate the prevalence of exposure to workplace bullying in a group of patients on sick leave or at risk of being sick listed due to CMD receiving treatment at an out-patient mental health clinic. A secondary aim was to examine the characteristics of patients currently or previously exposed to bullying at the workplace to determine the extent to which they differ from other patients presenting with CMD. The following research questions (RQs) will be examined: RQ1: What is the prevalence of exposure to bullying in patients referred to an outpatient clinic due to CMD? RQ2: Will patients exposed to bullying present with more psychiatric disorders, or higher levels of depressive symptoms, anxiety symptoms, subjective health complaints, alcohol use and lower levels of resilience compared to the patients not exposed to bullying? RQ3: Will patients exposed to bullying report higher levels of sick leave, and lower levels of work ability, job satisfaction and job preference (wishing to stay at their current job, change jobs or not work at all) compared to the patients not exposed to bullying?

## Materials and Methods

### Participants and Procedure

A total of 675 patients were included in the study. Data were collected in a naturalistic observational study in the project “The Norwegian studies of psychological treatments and work (NOR-WORK)” in an outpatient clinic at Diakonhjemmet Hospital in Oslo, Norway. The clinic offers cognitive or metacognitive therapy with a work focus and is a treatment option specialised for individuals with anxiety and depression who are on sick leave or at risk of exclusion from working life (for description of the treatment programme see, Gjengedal et al., 2020).

All patients included in the study were referred to the clinic by GPs due to mild-to-moderate anxiety and/or depressive disorders and were above the age of 18. The patients also had to be on sick leave or at risk of sick leave to be included in the study and the said treatment programme. Exclusion criteria were having severe mental disorders (e.g., bipolar), a high risk of suicide or substance abuse (including alcohol abuse). Data was obtained from June 2017 through January 2019. A total of 998 potential patients were assessed for inclusion in the study. Of these, 675 fulfilled the study inclusion criteria and consented to be a part of the study.

### Instruments

All participants completed a comprehensive paper-and-pencil questionnaire at intake including background variables in addition to a range of standardised instruments. Background variables included age, gender, education, and occupation.

#### Workplace Bullying

To measure exposure to bullying at the workplace we used the Short version of the Negative Acts Questionnaire (S-NAQ; Notelaers et al., 2019), which is a self-report measure consisting of nine items describing the most typical negative acts experienced by victim of bullying. Items are of a personal and social nature (e.g., ‘spreading gossip and rumours about you’), or a work-related nature (e.g., ‘persistent criticism of your work and effort’). The scale was scored on a scale from 1 (*never*) to 5 (*daily*) based on the last 6 months. The scale showed satisfactory reliability in the form of internal stability (Cronbach’s α = 0.88).

In addition to the S-NAQ, two single questions measuring self-labelled victimisation from workplace bullying at the patients current and previous workplaces was used. Response categories are coded: “No,” “Yes, once or twice,” “Yes, now and then,” “Yes, about once a week,” and “Yes, many times a week” (see also Nielsen et al., 2020).

Identified victims were then asked to complete the full version of the Negative Acts Questionnaire-Revised (NAQ-R; Einarsen et al., 2009) focussing on when their exposure was at its worst. This scale is the full version of the S-NAQ questionnaire. The NAQ-R consists of 22 items, where the items described negative acts directed at the individual (e.g., ‘being humiliated or ridiculed in connection with your work’) or at their work (e.g., ‘being withheld vital information’). The behaviour can be both direct (e.g., ‘openly attacking the victim verbally or physically’) and indirect (e.g., ‘social isolation’). Again, responses are given from 1 (*never*) to 5 (*daily*). The scale showed satisfactory reliability in the form of internal stability (Cronbach’s α = 0.91).

#### Psychiatric Disorders, Health, and Resilience

The Mini-International Neuropsychiatric interview (MINI; Sheehan et al., 1998) was used to identify psychiatric disorders in this population. MINI is a structured diagnostic interview assessing psychiatric disorders based on criteria of DSM-IV (American Psychiatric Association, 1994) and ICD-10 (World Health Organization, 1993). The interview is based on “yes” and “no” answers and covers 15 axis I disorders and 1 axis II disorder. This includes mood disorders (MDD and manic episodes), anxiety disorders (panic disorder, agoraphobia, social phobia, obsessive compulsive disorder, post-traumatic stress disorder, and generalised anxiety disorder), eating disorders (anorexia and bulimia), substance related disorders (alcohol and substances), psychotic disorders, and antisocial personality disorder. For the present study we used the Norwegian version of the MINI 6.0.0 (Leiknes et al., 2009).

The Beck Depression Inventory – II (BDI-II; Beck et al., 1996) was used as a self-reported measure of depressive symptoms. The scale consisted of 21 items measuring different affective and cognitive states, such as *sadness* and *guilt*, scored on a four-point Likert scale from 0 (*not at all*) to 3 (*severely – it bothered me a lot*), based on the patient’s state over the last 2 weeks. Based on sum scores, validated cut-off scores of ≤13 for minimal depressive symptoms, ≥ 14 for mild depressive symptoms, ≥ 20 for moderate depressive symptoms, and ≥29 for severe depressive symptoms were used for descriptive purposes. The scale showed satisfactory reliability in the form of internal stability (Cronbach’s α = 0.86).

The Beck Anxiety Inventory (BAI; Beck and Steer, 1990) was used as a self-reported measure of anxiety. The scale consisted of 21 items measuring anxiety symptoms scored on a four-point Likert scale from 0 (*not at all*) to 3 (*severely – it bothered me a lot*), based on the patient’s state over the last week. Based on sum scores, validated cut-off scores of ≤21 for low levels of anxiety symptoms, ≥ 22 for moderate levels of anxiety symptoms and ≥36 for potential concerning levels of anxiety symptoms were used for descriptive purposes. The scale showed satisfactory reliability in the form of internal stability (Cronbach’s α = 0.90).

The Subjective Health Complaints scale measured subjective somatic and psychological complaints over the last 30 days (SHC; Eriksen et al., 1999). The scale consisted of 29 items describing different common health complaints (e.g., musculoskeletal pain) scored on a four-point scale ranging from 0 (no complaints) to 3 (serious complaints). The scale provided five sub-categories all with satisfactory internal stability as measured with Cronbach’s alpha; gastrointestinal complaints (7 items, Cronbach’s α = 0.71), musculoskeletal complaints (8 items, Cronbach’s α = 0.77), pseudoneurology (7 items, Cronbach’s α = 0.68), allergy (5 items, Cronbach’s α = 0.52), and flu (2 items, Cronbach’s α = 0.64), in addition to a sum score for the total scale (29 items, Cronbach’s α = 0.84).

The Alcohol Use Disorders Identification Test AUDIT was used to screen for excessive drinking and alcohol use disorder (Saunders et al., 1993). This scale consisted of 10 items (e.g., “How often during the last year have you had a feeling of guilt or remorse after drinking”). Eight items were scored on a five-point scale and two items on a three-point scale. A score of eight or above indicated a strong possibility of harmful levels of alcohol consumption (Saunders et al., 1993). The scale showed satisfactory reliability in the form of internal stability (Cronbach’s α = 0.80).

To measure resilience the 33-item Resilience Scale for Adults (RSA; Hjemdal et al., 2001; Friborg et al., 2003, 2005) was used. Items (e.g., “My judgements and decisions”) were scored on a seven-point scale ranging from 1 (e.g., “I often doubt”) to 7 (e.g., “I trust completely”). The scale consists of six factors that in the current study was divided into two sub-dimensions: personal resilience (20 items, Cronbach’s α = 0.82) and interpersonal resilience (13 items, Cronbach’s α = 0.86), in addition to a sum score for the total scale (33 items, Cronbach’s α = 0.86).

#### Work

Employment status was measured using a single item with the options “work with no benefits,” “combined work and sick leave,” “full-time sick leave,” “unemployed,” “student,” “work assessment allowance,” “disability pension,” “neither work nor pension,” and “other.”

To measure work ability we used three items from the Work Ability Index (WAI; Tuomi et al., 1998). The first item was regarding sick leave the last 12 months with the options “no,” “0–2 months,” “3–6 months,” and “7–12 months.” Current work ability compared to life time best was scored on a scale from 1 (*no ability to work*) to 10 (*work ability at its best*), while work ability in relation to demands of the job, divided into physical demands and psychological demands, were scored on a scale from 1 (*very poor*) to 5 (*very good*).

Return to Work Self-Efficacy (RTW-SE; Lagerveld et al., 2010; Gjengedal et al., in press) was used as a self-reported measure of expectations concerning one’s own ability to return to and function well when working fully (e.g., being able to set boundaries, perform one’s work tasks, and being able to focus while at work). The scale consisted of 11 items (e.g., “I will be able to cope with setbacks”) scored on a six-point Likert scale from 1 (*totally disagree*) to 6 (*totally agree*). A higher score indicated a higher level of self-efficacy. The scale showed satisfactory reliability in the form of internal stability (Cronbach’s α = 0.89).

Job satisfaction was measured with one single question “Overall, how satisfied are you with your job?” scored on a five-point scale from 1 (*very satisfied*) to 5 (*very unsatisfied)*.

Job preferences was measured with one single question “If you could choose to have any job, what would you prefer?” with three response categories “*prefer not working at all*,” “*prefer a different job*,” and “*prefer the job I have today.*”

### Statistical Analysis

For descriptive purposes we calculated the severity of exposure to bullying among the self-labelled victims based on when the exposure had been at its worst measured using the NAQ-R (scores ranging from 22 to 110). This was calculated by using validated cut-off scores of 33 for occasionally exposed to bullying and 45 for severely exposed to bullying (see Notelaers and Einarsen, 2013). Further, we calculated the prevalence of exposure to bullying by using the cut-off values for the S-NAQ (scores ranging from 9 to 45). These were calculated based on the cut-off values for the NAQ-R divided by number of items included in the NAQ-R before multiplying with number of items included in the S-NAQ. For our analysis we divided the patients into not exposed to bullying (S-NAQ score 13 or lower) and exposed to bullying. Patients exposed to bullying were defined as patients scoring above the cut-off score for occasionally exposed to bullying (S-NAQ score 14 or higher).

Responses to the open-ended item regarding occupation were categorised using the Norwegian standard classification of occupations (STYRK-08; Statistics Norway, 2011), which is based on the International Standard Classification of Occupations 2008 (ISCO-08; International Labour Office, 2008). We did not test for significant differences between the occupations for the two patient groups due to too few cases in some occupational groups.

Preliminary analyses showed that all the variables were normally distributed except for SHC and AUDIT who were positively skewed. Thus, to explore the characteristics of the patients exposed to bullying and to investigate if they differ from other patients with CMD we employed Mann–Whitney *U*-tests and independent sample *t*-test for the continuous variables, and Chi-square tests were used for categorical variables. Additionally, we tested mean difference and Cohen’s *d* for the continuous variables. Statistical analyses were conducted with SPSS version 25.0 (IBM Corp, 2017). The significance level was set to α < 0.05.

### Ethical Considerations

The present study was conducted in accordance with the Helsinki Declaration and was approved by the Data Protection Office at Oslo University Hospital (ref. nr.: 2015/15606). All patients provided written informed consent.

## Results

### Background

The sample consisted of 70.5% women (*n* = 476) and had a mean age of 38.7 years (*SD* = 10.5; age ranged from 20 to 66 years). There was no significant difference between the patients exposed to bullying and the non-exposed patients for age or gender, nor for education or marital status (Table 1). Further, we found the largest difference in exposure to bullying among managers when investigating occupations with almost twice as many managers among those exposed to bullying.

**TABLE 1:** Background variables. Comparison between individuals exposed to bullying and non-targets tested with χ^2^ tests for gender, marital status, and education, and descriptive statistics for occupation (*N* = 634).

	**Exposed to bullying (*n* = 174)**		**Not bullied (*n* = 460)**		**χ *^2^***	**Effect size**	***p*-value**
	**%**	**(*n*)**	**%**	**(*n*)**			
Gender					1.32	0.05	0.250
Female	66.7	(116)	71.7	(330)			
Marital status					3.66	0.08	0.160
Single	32.0	(54)	31.7	(143)			
Married/cohabitating	58.6	(99)	63.0	(284)			
Separated/divorced	9.5	(16)	5.3	(24)			
Education					3.48	0.08	0.324
Primary school	1.8	(3)	2.0	(9)			
Upper secondary school	21.2	(36)	14.9	(68)			
Higher education 1–4 years	35.9	(61)	39.1	(178)			
Higher education > 4 years	41.2	(70)	44.0	(200)			
Occupation							
Managers	20.1	(35)	11.5	(53)			
Professionals	44.3	(77)	58.3	(267)			
Technicians and associate professionals	10.3	(18)	14.6	(61)			
Clerical support workers	7.5	(13)	2.6	(12)			
Service and sales workers	11.5	(20)	10.7	(48)			
Skilled agricultural, forestry and fishery workers	0.6	(1)	0.4	(2)			
Craft and related trade workers	3.4	(6)	1.1	(5)			
Plant and machine operators, and assemblers	0.6	(1)	0.2	(1)			
Elementary occupations	0.0	(0)	0.0	(0)			
Armed forces and unspecified	1.1	(2)	2.4	(11)			

*For 2 × 2 tables phi coefficient is reported, while Cramer’s V is reported for tables larger than 2 × 2. Professionals include occupations such as engineering, health, and teaching professions.*

### Workplace Bullying

Many patients reported exposure to bullying, and one fourth of the sample (25.8%) could be classified as being subjected to systematic exposure to bullying – defined as scoring above the cut-off score for occasionally exposed to bullying using S-NAQ scores (Table 2). The prevalence using self-labelling for both current and previous workplace were lower than for the S-NAQ, which is to be expected. However, among targets identified by self-labelling at a current or previous workplace (*n* = 193), 33.7% could be classified as being severely exposed to workplace bullying.

**TABLE 2:** Prevalence of exposure to bullying at the workplace measured with Short-Negative Acts Questionnaire (S-NAQ) and self-labelling. Severity of exposure to bullying measured with Negative Acts Questionnaire-Revised (NAQ-R) (*N* = 661).

	**%**	***(n)***	***M***	**(SD)**
S-NAQ (last 6 months)			12.8	(5.2)
Not bullied	68.1	(460)		
Occasionally exposed to bullying	14.5	(98)		
Severe exposure to bullying	11.3	(76)		
Self-labelling (current workplace)				
No	83.4	(563)		
Yes, once in a while	7.3	(49)		
Yes, sometimes	4.6	(31)		
Yes, once a week	0.9	(6)		
Yes, several times a week	1.3	(9)		
Self-labelling (previous workplace)				
No	83.4	(563)		
Yes, over a short time period	10.5	(71)		
Yes, over a long time period	4.0	(27)		
NAQ-R (exposure to bullying at its worst)			45.7	(14.7)
Not bullied	12.7	(21)		
Occasionally exposed to bullying	27.7	(46)		
Severe exposure to bullying	33.7	(56)		

### Psychiatric Disorders, Health, and Resilience

Major depressive disorder and generalised anxiety disorder were the most common diagnosis among the targets of bullying. The prevalence of on-going MDD was significantly larger among patients exposed to bullying compared to the non-exposed patients (Table 3), while no significant differences between the patient’s groups were seen for other psychiatric disorders evaluated with the MINI. These results from the MINI were also reflected in the scores for depressive and anxiety symptoms (measured with BDI-II and BAI). The patients exposed to bullying reported significantly more depressive and anxiety symptoms compared to the non-exposed patients (Table 4). According to predefined cut-off values for depressive symptoms as measured with BDI-II, 45.2% of the patients exposed to bullying reported severe levels of depressive symptoms (BDI-II score 29 or higher) in comparison to 34.7% of the non-exposed patients [*X*^2^(3) = 9.75, *p* = 0.021, Cramer’s *V* = 0.13]. Further, 7.6% among the patients exposed to bullying could be classified as having severe levels of anxiety symptoms measured with BAI (BAI score 36 or higher) compared to 4.9% among the non-exposed patients [*X*^2^(2) = 11.51, *p* = 0.003, Cramer’s *V* = 0.14]. Additionally, patients exposed to bullying reported significantly lower resilience scores (Table 4), as well as reporting more subjective health complaints, and a higher alcohol consumption compared to the non-exposed patients (Table 5).

**TABLE 3:** Psychiatric disorders as measured with the Mini-International Neuropsychiatric Interview (MINI). Comparison between individuals exposed to bullying and non-targets tested with χ^2^ tests (*N* = *634)*.

	**Exposed to bullying (*n* = 174)**		**Not bullied (*n* = 460)**		**χ *^2^***	***p*-value**	**Effect size**
	**%**	**(*n)***	**%**	**(*n*)**			
Diagnosis assessment (MINI)							
Major depressive disorder (on going)	70.7	(123)	60.0	(276)	5.73	0.017	0.10
Major depressive disorder (previous)	23.0	(40)	26.1	(120)	0.49	0.485	−0.03
Major depressive disorder (reoccurring)	14.4	(25)	15.2	(70)	0.02	0.789	−0.01
Agoraphobia	17.2	(30)	12.8	(59)	1.69	0.194	0.06
Generalised anxiety disorder	46.0	(80)	46.3	(213)	0.00	1.000	0.00
Panic disorder	23.0	(40)	23.9	(110)	0.02	0.889	−0.01
Post-traumatic stress disorder	2.3	(4)	2.8	(13)	0.01	0.927^a^	−0.02
Social phobia	17.8	(31)	14.8	(68)	0.67	0.414	0.04

*For 2 × 2 tables phi coefficient is reported as effect size. ^*a*^ One cell had an expected cell count less than 5. Exact p value (Fischer’s exact test significance) was used.*

**TABLE 4:** Depression, anxiety, and resilience. Comparison between individuals exposed to bullying and non-targets tested with independent-*t*-tests (*N* = 634).

	**Exposed to bullying (*n* = 174)**		**Not bullied (*n* = 460)**		***t−*Value**	***p*−value**	**Mean difference**	**95% Confidence interval of the difference**		***Cohen’s d***
	**Mean**	**(SD)**	**Mean**	**(SD)**				**Lower**	**Upper**	
Depressive symptoms (BDI-II), 0–63	28.84	(9.45)	25.17	(8.52)	4.59	<0.001	3.67	2.10	5.24	0.42
Anxiety symptoms (BAI), 0–63	20.79	(10.17)	18.21	(9.67)	2.73	0.007	2.58	0.72	4.44	0.26
Resilience, 33–231	140.06	(23.76)	145.33	(24.00)	–2.29	0.023	–5.27	–9.79	–0.75	−0.22
Personal, 20–140	73.79	(15.93)	76.13	(16.84)	–1.54	0.124	–2.35	–5.34	0.65	−0.14
Interpersonal, 13–91	66.66	(13.03)	69.06	(12.66)	–2.02	0.044	–2.41	–4.74	–0.07	−0.19

**TABLE 5:** Health and alcohol use. Comparison between individuals exposed to bullying and non-targets tested with Mann–Whitney *U*-tests (*N* = 634).

	**Exposed to bullying (*n* = 174)**		**Not bullied (*n* = 460)**		***U*-value**	***z*-value**	***p*-value**	**Effect size**
	**Median**	**(SD)**	**Median**	**(SD)**				
Subjective health complaints, 0–87	25	(11.56)	21	(9.74)	22449.50	− 3.83	<0.001	−0.17
Musculoskeletal, 0–24	7	(5.12)	6	(4.31)	31801.00	− 1.10	0.273	−0.05
Pseudoneurology, 0–21	10	(3.77)	10	(3.61)	32769.50	− 2.16	0.031	−0.09
Gastrointestinal, 0–21	4	(3.65)	3	(3.32)	27512.00	− 4.19	<0.001	−0.17
Allergy, 0–15	2	(2.36)	1	(2.01)	33472.00	− 1.84	0.066	−0.07
Flu, 0–6	1	(1.40)	0	(1.26)	33215.00	− 2.55	0.011	−0.10
Alcohol use (AUDIT), 0–42	5	(5.00)	4	(3.91)	31952.50	− 2.04	0.042	−0.08

*For Mann–Whitney *U*-tests *r* was reported as effect size.*

### Work

There was a statistically significant difference in employment status between the non-bullied and bullied. Almost twice as many patients exposed to bullying were on full-time sick leave compensation compared to the non-exposed patients, while there was a larger percentage of the non-exposed patients who combined work and sick leave, that is being partially on sick leave (Table 6). No significant difference existed between the groups when examining their self-reported sick leave over the last 12 months. Over all, the patients exposed to bullying reported significantly poorer self-reported current work ability compared to life time best (scale from 1 to 10, *M* = 3.99, *SD* = 2.67) as compared to the non-exposed patients [*M* = 5.15, *SD* = 2.46; *t*(621) = −5.07, *p* < 0.001]. The magnitude of the differences in the means (mean difference = −1.15, 95% CI: −1.60 to −0.71) was in the medium effect size range (Cohens *d* = −0.46). Regarding work ability, targets of bullying reported a significantly poorer ability to handle both the psychological and physiological demands of their job as compared to other patients.

**TABLE 6:** Sick leave, work ability and job satisfaction. Comparison between individuals exposed to bullying and non-targets tested with χ^2^ tests (*N* = 634).

	**Exposed to bullying (*n* = 174)**		**Not bullied (*n* = 460)**		**χ *^2^***	**Effect size**	***p*-value**
	**%**	**(*n*)**	**%**	**(*n*)**			
Employment status					24.06	0.20	<0.001
Work with no benefits	40.0	(68)	49.6	(222)			
Combined work and sick leave	20.6	(35)	30.1	(135)			
Full-time sick leave	39.4	(67)	20.3	(91)			
Sick leave (last 12 months)					4.03	0.08	0.258
No	29.8	(48)	30.8	(131)			
0–2 months	46.6	(75)	46.6	(198)			
3–6 months	21.1	(34)	16.7	(71)			
7–12 months	2.5	(4)	5.9	(25)			
Job satisfaction					80.47	0.36	<0.001
Very satisfied	2.4	(4)	16.4	(73)			
Satisfied	20.7	(34)	41.4	(184)			
Neutral	30.5	(50)	24.5	(109)			
Unsatisfied	26.2	(43)	13.1	(58)			
Very unsatisfied	20.1	(33)	4.5	(20)			
Job preference					46.11	0.28	<0.001
Prefer not working at all	5.7	(9)	2.6	(11)			
Prefer a different job	73.9	(116)	45.7	(192)			
Prefer the job I have today	20.4	(32)	51.7	(217)			
Work ability in relation to job demands							
Psychological demands					40.10	0.25	< 0.001
Very good	4.7	(8)	4.9	(22)			
Good	5.8	(10)	19.0	(86)			
Moderate	29.2	(50)	40.8	(185)			
Poor	44.4	(76)	29.1	(132)			
Very poor	15.8	(27)	6.2	(28)			
Physiological demands					18.85	0.17	0.001
Very good	28.7	(49)	39.1	(176)			
Good	34.5	(59)	38.0	(171)			
Moderate	21.6	(37)	17.1	(77)			
Poor	12.9	(22)	4.7	(21)			
Very poor	2.3	(4)	1.1	(5)			

*For 2 × 2 tables phi coefficient is reported, while Cramer’s V is reported for tables larger than 2 × 2.*

Further, patients exposed to bullying reported significantly lower return to work self-efficacy (RTW-SE) (scale from 1 to 6, *M* = 3.03, *SD* = 0.89) than the non-exposed patients [*M* = 3.32, *SD* = 0.98; *t*(629) = −3.42, *p* = 0.001]. The magnitude of the differences in the means (mean difference = −0.29, 95% CI: −0.46 to −0.12) was within the medium effect size range (Cohens *d* = 0.30).

Targets of bullying reported significantly lower job satisfaction, and a majority among them reported that they would prefer another job than the one they have today, a significant higher proportion than among the rest of the patients. However, very few reported not wanting to work at all.

## Discussion

The results from the present study indicate a high prevalence of exposure to bullying in patient populations with CMD seeking treatment. As many as one in four had been subjected to systematic exposure to bullying at work in the present sample. Although patients exposed to workplace bullying come from all kinds of professions and industries, descriptive analysis indicated that there were almost twice as many managers among the exposed patients as compared to non-exposed patients. Previous findings indicate that bullying is not more prevalent among managers (Skogstad et al., 2008). The present findings may thus indicate that when managers seek treatment for mental disorders, they are more likely to do so because of exposure to bullying. We should take note of the fact that patients seeking such treatment may be managers with a history of bullying. Further, the prevalence of major depressive disorders diagnosed with the psychiatric interview (MINI) were higher in the patients exposed to bullying than for the patients not exposed to bullying. Consistent with this, they also reported higher levels of depressive and anxiety symptoms, more subjective health complaints, and higher levels of alcohol consumption than patients not exposed to bullying. In addition to reporting lower job satisfaction and lower work ability, as many as 74% reported that they would prefer another job than the one they have today. Hence, for these patients, return to work after sick leave is more about returning to working life than about recovering into ones existing job, which probably means facing their predicament again.

The prevalence of systematic exposure to bullying in this patient sample is quite high compared to the general population, both when examining exposure to bullying and perceived victimisation from bullying (self-labelling). The prevalence in the Norwegian general population ranges from 4.6% (self-labelling) to 14.3% (exposure to at least one negative act a week) (Nielsen et al., 2009) compared to 14.1 and 25.8%, respectively in the present study. As prevalence of bullying is generally low in Norway (see also Van de Vliert et al., 2013), even higher proportions may be found in other countries. This prevalence is also quite high compared to healthcare workers in Europe, a sector known for having high prevalence of bullying, where a systematic review by Lever et al. (2019) found a mean prevalence of 18.4%. In a sample of patients receiving psychiatric care for workplace traumas in out-patient clinics in Turkey, as many as 43.3% reported exposure to workplace bulling (Tatar and Yüksel, 2019). Prevalence rates estimated from measure of exposure to negative acts such as the NAQ-R or S-NAQ often varies between 10.0 and 17.0% in other countries (Zapf et al., 2020). It is also worth pointing out that 33.7% self-labelled as being or having been a victim of severe bullying in the present study’s patients sample, while the same can only be said for about 6.8% in the Norwegian general population (Nielsen et al., 2009). Thus, these findings provide support to our presumptions that patients on risk for or on sick leave seeking treatment for CMD would have a high prevalence of exposure to bullying, both currently when seeking treatment as well as a part of their occupational history.

Patients exposed to bullying, reporting significantly more health complaints than other patients. As many as 70.7% presented with on-going MDD in accordance with criteria from DSM-IV. The prevalence was significantly higher compared to the non-exposed patients (60.0%). These results are similar to findings from a study performed on out-patient clinics in Turkey, where 78.5% of patients exposed to bullying could be diagnosed with MDD in accordance with criteria from DSM-IV-TR (Tatar and Yüksel, 2019).

Furthermore, severity of symptoms of depression (measured with BDI-II) and anxiety (measured with BAI), as well as pseudoneurology related complaints (measured with SHC), were also significantly higher compared to the other non-exposed patients and to the general Norwegian population (Statistics Norway, 2012; Indregard et al., 2013; Kjærgaard et al., 2014). Considering the detrimental effects caused by being exposed to bullying found in previous studies (e.g., Lahelma et al., 2012; Kostev et al., 2014; Lo Presti et al., 2019), the high levels of depressive and anxiety symptoms among these patients are not surprising. These findings are in accordance with previous research where exposure to workplace bullying have been associated with an increase in both depressive and anxiety symptoms (e.g., Verkuil et al., 2015; Lo Presti et al., 2019). When it came to subjective health complaints the exposed group particularly reported higher levels of gastrointestinal complaints. The high comorbidity of health complaints may be explained in the framework of stress theories like The Cognitive Activation Theory of Stress (CATS; Ursin and Eriksen, 2004). It suggests that individuals who has been exposed to threatening behaviour with the experienced lack of coping, will develop an increased sensitisation due to repeated exposure to the stimulus (e.g., systematic exposure to bullying) (Ursin and Eriksen, 2010; Ursin, 2014). Due to attentional bias the individual’s thoughts and information regarding the bullying will be prioritised, thereby causing a perseverative cognition, manifested in rumination and worrying, which may further lead to a prolonged activation (Brosschot et al., 2006). This may again lead to somatic complaints and diseases by causing increased activation via the immune, endocrine, cardiovascular, and the autonomic nervous system (Brosschot et al., 2006). This line of argument is consistent with these patients scoring higher on gastrointestinal complaints, which could be explained by the enhanced activation of the autonomic nervous system causing strain on their internal organs, such as the gastrointestinal tract. While the increased flu symptoms could potentially be explained by the sustained activation having a negative effect on the immune system.

Based on CATS, having higher levels of resilience would help the individual cope when exposed to stressors (e.g., exposure to bullying) and protect against sustained activation. Yet, Zapf and Einarsen (2005) argues strongly that exposure to ongoing bullying will eventually lead to loss of coping resources, as also shown empirically in a five year longitudinal study among nurses where those targetted over many years showed a significant reduction in the personality trait hardiness, a trait similar to the concept of resilience (Hamre et al., 2020). In the present data this may be reflected in our finding indicating that patients exposed to bullying have lower levels of resilience, which also is an explanation why the bullied display more health complaints than the other non-exposed patients.

Earlier studies has found that workplace bullying is associated with problematic levels of alcohol consumption (Nielsen et al., 2018), which may be caused by elevated negative work rumination, a mechanism found to relate to high consumption of alcohol (Frone, 2015). Our findings indicated that the participants in this study did not have alcohol related problems although the exposed patients did score significantly higher than the non-exposed patients. It is thus not a level of concern in the current dataset, but it is still a difference that may be clinically useful to have in mind.

In accordance with previous studies the high prevalence of depression, anxiety, and subjective health complaints found for the patients exposed to bullying were considerable compared to the non-exposed patients, thus supporting our presumptions.

The exposed group evaluated their relation to work more negatively which also is in line with established consequences of workplace bullying (Nielsen and Einarsen, 2012), and may even be expected in a group exposed to such a severe stressor at their workplace. These patients, consistent with previous studies (Olsen et al., 2017), evaluate their own work ability as being low. The fact that these patients evaluate their own work ability and their ability to handle the psychological demands of their work poorer than other patients, could be part of the reason why there was almost twice as many on full-time sick leave among the patients exposed to bullying compared to the non-exposed patients. This is consistent with previous findings in both Norway (Nielsen et al., 2016) and other European countries (Niedhammer et al., 2012) where workplace bullying has been established as a major risk factor for sick leave. There were almost twice as many among the bullied patients on full-time sick leave compared to the other patients in the present study. The high proportion of bullied patients currently on full-time sick leave may indicate that sick leave is a way of coping with the adversity of the bullying limiting the contact with the bully and related adverse situations. However, the lack of difference between the patient groups when examining their sick leave over the last 12 months in the present study, may relate to other findings showing that employees exposed to bullying have higher sickness presenteeism than non-bullied employees (e.g., Høgh et al., 2011). This could be a plausible explanation for why there is not a difference in sick leave over time. They stay at work as long as possible and when they do not cope anymore, full-time sick leave is the only option. The high amount of these patients on full-time sick leave can also be reflected in their low RTW-SE scores, which measures the individuals perceived ability and confidence regarding their ability to handle expected demands when returning to work (Lagerveld et al., 2010).

Workplace bullying has consistently been associated with lowered job satisfaction (Arenas et al., 2015; Olsen et al., 2017), which is also in line with our findings. Thus, our findings support the notion that workplace bullying could be seen as a severe work stressor associated with high levels of psychological distress and reduced well-being at work. This is further exemplified by the fact that as many as 74% of the patients exposed to bullying said they preferred another job than the one they had. It is however important to note that only 5.7% of these patients preferred to not work at all. In comparison about half of the non-exposed patients preferred another job and the other half preferred to stay in their current job. All in all, this may indicate that the problems of these patients are actually rooted in their job situation more than what is typical for other patients presenting with CMD.

### Implications

The results from the present study highlights the role of workplace bullying in patients seeking treatment for mental disorders and who are at risk of exclusion from working life. First of all, many patients seeking mental health treatment will present with an on-going or a history of victimisation from workplace bullying. It is important to note, however, that these are generally motivated to stay in working life, but do not wish to stay at their current job. They also have higher levels of mental health complaints than non-exposed patients. Thus, it could be of value to identify patients exposed to bullying in outpatient clinics addressing these differences in the treatment. If not addressed there is a risk of sick leave for these patients and subsequently a risk of expulsion from the workplace and potentially working life itself. It seems to be central to both identify those that have been exposed to bullying among those that seek treatment for CMD, and also develop good procedures for altering their employment. Furthermore, when considering that as many as one out of four individuals on sick leave or at risk due to CMD seeking treatment are exposed to bullying, and that these individuals seem to have more severe symptoms and almost twice as many are on full-time sick leave when compared to the other patients, suggests that bullying can become a substantial cost for employers, and the society at large. It should therefore be a focus on implementing intervention programmes in organisations as a preventive measure for workplace bullying.

### Strengths and Limitations

Some important strengths and limitations of the study must be addressed. In this respect it is worth mentioning that the study is compiled of a large number of well-established and psychometrically sound instruments. Furthermore, to measure mental health related complaints in this study we used a well-known clinical interview and frequently used self-report questionnaire to assess levels of symptoms (MINI, BDI-II, BAI, and SHC). Additionally, to measure the prevalence of exposure to workplace bullying we used both self-labelling as a victim of workplace bullying and self-report of exposure to bullying, in line with recent recommendations (Nielsen et al., 2020). However, it should be mentioned that self-reported measures are not considered to be as reliable as objective measures. Nevertheless, most studies investigating workplace bullying examines perceived exposure to bullying as one might argue that workplace bullying is a concept that is subjective in its very nature.

Due to multiple comparisons on a large number of outcome variables the results from the analyses comparing patients exposed to bullying and non-exposed patients should be interpreted with some caution. However, most of the differences in our results had a significance level of *p* < 0.01. Another possible limitation is the studies cross-sectional design, which does not account for causal relationships between the study variables. In addition, only one clinic was included in the study, which may limit the generalisability of the study results to outpatient clinics at large.

## Conclusion

The results from the present study provide an important insight into a vulnerable group of patients who are at risk of losing their foothold in working life. While being exposed to bullying at the workplace may result in poor health, being exposed to this type of negative behaviours can also have severe negative consequences for the individuals work ability and job satisfaction. This study contributes to the literature by providing evidence that patients exposed to bullying seem to be overrepresented among patients with CMD and they seem to have more severe health complaints compared to other patients with CMD. This in addition to experiencing more negative work outcomes and almost twice as many being on full-time sick leave. Thus, this sheds a light on a problem that should be addressed in clinical settings to improve the treatment of these patients so to avoid potential detrimental outcomes for the individual when this issue is not addressed. Future studies should build on this by examining causal relationships and investigating if and to what extent psychological treatment have a similar curative effect on those that are exposed to bullying as compared to other patients.

## Data Availability Statement

The datasets presented in this article are not readily available because the participants have not consented to distribution of data outside the studies conducted at the clinic and its specific conditions regarding confidentiality, privacy protection and data handling approved by the Data Protection Office at Oslo University Hospital and described to the participants. Requests to access the datasets should be directed to SA, Sarah.Aarestad@uib.no.

## Ethics Statement

The studies involving human participants were reviewed and approved by The Data Protection Office at Oslo University Hospital (ref. nr.: 2015/15606). The patients/participants provided their written informed consent to participate in this study.

## Author Contributions

SHA, AH, SVE, and OH conceived and designed the study. RGHG, KO, MH, KS, MTB, and OH were responsible for data collection. SHA analysed the data. SHA, AH, SVE, OH, and KO contributed in the interpretation of results. SHA wrote the first original draft. AH, SVE, OH, RGHG, KO, KS, MTB, and MH were involved in reviewing, and editing of the manuscript. All authors read and approved the final manuscript.

## Conflict of Interest

The authors declare that the research was conducted in the absence of any commercial or financial relationships that could be construed as a potential conflict of interest.
